# The Effect of Dipping Tobacco on Pulse Wave Analysis among Adult Males

**DOI:** 10.1155/2020/7382164

**Published:** 2020-10-19

**Authors:** Areeg E. Elemam, Nisreen D. Omer, Neima M. Ibrahim, Ahmed B. Ali

**Affiliations:** ^1^Al Neelain University, Faculty of Medicine, Khartoum, Sudan P.O. Box 12702; ^2^University of Khartoum, Faculty of Medicine, Khartoum, Sudan P.O. Box 102; ^3^Almaarefa University, Faculty of Medicine, Riyadh, Saudi Arabia; ^4^University of Khartoum, Faculty of Nursing Sciences, Khartoum, Sudan P.O. Box 102; ^5^Cardiology Department, Al-Shaab Teaching Hospital, Khartoum, Sudan

## Abstract

**Background:**

The current study investigated the effect of dipping tobacco (DT) use on arterial wall stiffness which is a known marker of increased risk of cardiovascular events.

**Methods:**

A case-control study which included 101 adult males was carried out in Al-Shaab Teaching Hospital. Blood pressure and pulse wave analysis parameters were recorded in 51 DT users (study group) before and after 30 minutes of placing tobacco and in 50 nontobacco users (control group). Anthropometric measurements were collected using data collection sheet. Data were entered into a computer and analyzed by using the software Statistical Package for the Social Sciences (SPSS) version 20 (SPSS Inc., Chicago, IL, USA).

**Results:**

At baseline measurements, heart rate (HR) was significantly lower in the study group compared to the control group (66.15 ± 9.21 vs. 72.87 ± 10.13 beats/min; *P* value ≤ 0.001). Subendocardial viability ratio (SEVR) was significantly higher in the study group compared to the control group (203.44 ± 30.34 vs. 179.11 ± 30.51%; *P* value ≤ 0.001). Acute effects of DT compared to pretobacco dipping showed significant increase in HR (72.50 ± 10.89 vs. 66.15 ± 9.21 beats/min; *P* value ≤ 0.001) and significant decrease in augmentation pressure (AP) (4.30 (2.30-8.00) vs. 3.30 (0.60-6.3) mmHg; *P* value ≤ 0.001), ejection duration (ED) (271.65 ± 19.42 vs. 279.53 ± 20.47 ms; *P* value ≤ 0.001), and SEVR (187.11 ± 29.81 vs. 203.44 ± 30.34; *P* value ≤ 0.001). Linear regression analysis for AP predictor showed that only HR and AIx@75 affect and predict the values of AP (Beta ± SE; −0.242 ± 0.019, *P* value ≤ 0.001; 0.685 ± 0.014, *P* value ≤ 0.001).

**Conclusions:**

Long-term use of DT was not associated with permanent changes in heart rate and blood pressure. Acute tobacco dipping caused an acute increase in heart rate and oxygen demands of myocardium.

## 1. Introduction

Smokeless tobacco (ST) use is rising worldwide. Globally, more than six million disability-adjusted life-years were lost and more than 250.000 deaths occurred in 2010 due to ST consumption [1]. In Sudan, 34% of the adult population use a dipping tobacco (DT), a form of ST and locally named *toombak* [2]. Previous studies demonstrated that long-term ST product use was associated with a modest risk of fatal myocardial infarction (MI), hypertension, and metabolic syndrome [3, 4]. The adverse effects of ST on the cardiovascular system are mainly attributed to nicotine constituent. Although there are other constituents present with nicotine and vary from country to other [5, 6], nicotine has a range of cardiovascular effects which mimic the effect of the sympathetic nervous system, acutely increasing blood pressure, heart rate, and the strength of the heart's contractions. The consequences of these effects are increasing the demand of cardiac muscles for oxygen and nutrients [5, 7]. One-time use of ST had been reported to lead to significant transient increase in heart rate, central aortic pressures, and aortic augmentation index normalized to a fixed heart rate of 75 bpm (AIx@75) [8]. Chronic ST users therefore may be subject to continuous elevation of heart rate, central pressures, and left ventricular work and have an increased cardiovascular risk. Nicotine increases cardiovascular risk through many mechanisms. Nicotine promotes inflammation, thus potentially contributing to atherogenesis [9]. Moreover, nicotine directly affects blood vessels and leads to endothelial dysfunction by multiple reported mechanisms. Some studies reported decreased availability of nitric oxide in arteries and veins as a response to nicotine [10–13]. Nicotine was detected to increase endothelin-1 production by endothelial cells [14]. Impaired venous and arterial endothelium-dependent dilation had been demonstrated also in tobacco consumers [15]. The risk of cardiovascular diseases associated with the use of new and emerging tobacco products, such as electronic cigarettes, hookah, heat-not-burn products, and *toombak*, remains unclear. This may be due to the lack of knowledge of the mechanisms by which these products affect cardiovascular health. Such cardiovascular injury could be assessed at several levels by many methods. Vascular dysfunctions induced by nicotine could be evaluated by measuring endothelial function (flow-mediated dilation), platelet aggregation, arterial stiffness, endothelial microparticles, and platelet-leukocyte aggregates [16]. The study of the association of these biomarkers with the use of new and emerging tobacco products could provide insights into mechanisms by which they induce cardiovascular harm. Pulse pressure in central arteries is known as an independent predictor of adverse cardiovascular events [17–21]. Arterial wall stiffness can be assessed noninvasively through pulse wave analysis (PWA). PWA now became one of the tools for assessment of vascular integrity and its effect on disease entities [22, 23]. It is now possible to record the pulse wave from the radial or carotid artery, to synthesize the ascending aortic pulse waveform, identify ejection duration, and generate indices of arterial wall stiffness (augmentation pressure and augmentation index) [22]. Although *toombak* users are widely distributed in Sudan, there is a lack of studies that clarify the cardiovascular hazards of *toombak* use. Therefore, the aim of this study was to determine the effects of regular and chronic use of DT on arterial wall stiffness indicated by central aortic blood pressure and wave reflection characteristics using PWA. Such information is of paramount importance for healthcare providers, caregivers, health educators, and policy makers.

## 2. Materials and Methods

A case-control study was carried out in Al-Shaab Teaching Hospital, Khartoum, Sudan, during the period from October to December 2018. The participants of the study group were 51 healthy adult males aged between 18 and 45 years using dipping tobacco only and scored 5 or more in the Fagerstrom nicotine dependence-smokeless tobacco questionnaire (FNDT). All the participants were normotensive (<140/90 mmHg), not known to have any cardiovascular disease and chronic illnesses, and not taking any medications. The participants of the control group were 50 healthy adult males aged between 18 and 45 years and never used any kind of tobacco. For both groups, objectives and procedure of the research were explained and then written informed consent was obtained from each participant. Participants of the study group were asked to refine themselves from tobacco dipping overnight before reporting to the study at early morning.

### 2.1. Age and Anthropometric Measurements

Weight and height were recorded for all participants in the study and control groups. Weight was measured using Click on 6 in 1 Electronic Body Fat Scale A1412, and height was measured by the RGZ120, China, standometer. Body mass index (BMI) was calculated using the following formula: BMI (kg/m^2^) = weight (kg)/height (m^2^).

### 2.2. Blood Pressure Measurement

After resting in sitting position for 10 minutes, brachial blood pressure was measured by the auscultatory method using an Aneroid Sphygmomanometer (Certified CE 0483, Tokyo, Japan). The mean of three readings was recorded.

### 2.3. Pulse Wave Analysis

PWA was recorded from the radial artery by a tonometer (SphygmoCor Pulse Wave Analysis System, Software version 8.2, AtCor Medical, model: EM3, Sydney, Australia). Then, the volunteers of the study group were asked to place their usual dose of dipping tobacco, and after 30 minutes, brachial blood pressure and PWA were recorded (postdipping tobacco). Subjects were instructed not to consume anything during data collection. All PWA workup was conducted in Al-Shaab Teaching Hospital, Cardiology Department.

### 2.4. Statistical Analysis

Data were entered into a computer and analyzed by using the software Statistical Package for the Social Sciences (SPSS) version 20 (SPSS Inc., Chicago, IL, USA). The data were expressed as mean + /−SD if normally distributed or median (25^th^–75^th^ interquartile) if abnormally distributed. Independent Student's *t*-test was used to compare the study and control groups. Paired *t*-test was used to compare pre- and postdipping tobacco use for the study group. The Mann–Whitney *U* test was used to compare the study and control groups when data were abnormally distributed while the Wilcoxon signed-rank test was used to compare pre- and postdipping tobacco use for the study group when data were abnormally distributed. *P* value ≤ 0.01 was considered statistically significant.

## 3. Results

### 3.1. Participants' Basic Characteristics

This study included a total of 101 healthy adult males; 51 of them were dipping tobacco users and scored ≥5 in the Fagerstrom nicotine dependence-smokeless tobacco questionnaire (FNDT) and 50 are nontobacco users as the control group. Both groups were well matched in their basic characteristics, age, and BMI (Table 1).

### 3.2. Central and Peripheral Hemodynamic Parameters of the Study Group Pretobacco Dipping vs. the Control Group

The comparison revealed that HR was significantly lower in the study group compared to the control group (66.15 ± 9.21 vs. 72.87 ± 10.13 beats/min; *P* value ≤ 0.001). SEVR was significantly higher in the study group compared to the control group (203.44 ± 30.34 vs. 179.11 ± 30.51%; *P* value ≤ 0.001) (Table 2).

### 3.3. Linear Regression Analysis between AP and Other Clinical and PWA Parameters in Pretobacco Dipping

The linear regression model for AP showed that only HR and AIx@75 affect and predict the values of AP (Beta ± SE; −0.242 ± 0.019, *P* value ≤ 0.001; 0.685 ± 0.014, *P* value ≤ 0.001) (Table 3).

### 3.4. Results of PWA in the Study Group Posttobacco Dipping vs. the Control Group

There were no significant differences in central and peripheral hemodynamic and PWA parameters of the study group after 30 minutes of dipping tobacco in comparison with the control group (Table 4).

### 3.5. Results of PWA Parameters in the Study Group Pretobacco Dipping vs. Posttobacco Dipping

Comparison of central and peripheral hemodynamic and PWA parameters pre- and posttobacco dipping showed that dipping tobacco was associated with a significant decrease in AP (4.30 (2.30-8.00) vs. 3.30 (0.60-6.3) mmHg; *P* value ≤ 0.001), ED (279.53 ± 20.47 vs. 271.65 ± 19.42 ms; *P* value ≤ 0.001), and SEVR (203.44 ± 30.34 vs. 187.11 ± 29.81; *P* value ≤ 0.001) and a significant increase in heart rate (66.15 ± 9.21 vs. 72.50 ± 10.89 beats/min; *P* value ≤ 0.001) (Table 5).

## 4. Discussion

Over the past decades, research has led to the identification of a wide range of cardiovascular disease risk factors associated with cigarette smoking as well as its biomarkers. The cardiovascular disease risk and its biomarkers associated with the use of ST and emerging new tobacco products remain unclear. Arterial wall stiffness is an important earlier marker for devastating adverse effect of DT on cardiovascular morbidity. Pulse wave analysis (PWA) is one of the noninvasive methods used to measure arterial stiffness.

The current study is aimed at answering the question about the effect of DT on PWA parameters in normal subjects who use DT regularly compared to normal subjects who never use any sort of tobacco. The major finding in this study is that the refining from dipping tobacco overnight was associated with a significant decrease in heart rate. This decrement in heart rate may be owed to overnight abstinence from dipping tobacco, as bradycardia is a known sign of nicotine withdrawal [24]. Different subtypes of acetylcholine nicotinic receptors have been reported to mediate the effect of nicotine withdrawal in mice [25–27]. Do nicotinic acetylcholine receptors mediate bradycardia in nicotine withdrawal, a question that needs further research. In contradiction, acute tobacco dipping significantly increased heart rate. Many studies detected a similar result [28–30]. The level of nicotine produced by smokeless tobacco is higher than that seen with smoking and causes similar sympathetic neural stimulation and acute cardiovascular effects [31]. Smoke and smokeless tobacco induce increased heart rate whether it is mediated by increased norepinephrine released locally from adrenergic axon terminals within the heart or by increased release of catecholamine. This needs to be solidly determined as studies reported controversial findings [7, 32, 33].

In this study, regular use of DT does not cause constant increase in heart rate. It is the acute exposure to DT that increases heart rate. This observation is consistent with a previous study which reported that various forms of smokeless tobacco (mainly snuff and chewing tobacco) caused an immediate increase in heart rate, but regular users of smokeless tobacco do not have permanent changes in heart rate when not exposed to tobacco [28].

In this study, BP, AP, and AIx@75 did not differ significantly between DT users and nonusers. However, acute tobacco dipping significantly increased heart rate and decreased AP, SEVR, and ED, but there was no significant change in AIx@75. Previous studies reported conflicting results regarding BP changes in smokeless tobacco users. Increase of BP, AP, and AIx@75 has been documented with smokeless tobacco, and even single-time use of ST was reported to increase systolic blood pressure, AP, and AIx@75 [8]. However, some studies reported no significant differences in BP between ST users and nonusers [6, 34]. This can be explained by the presence of different forms of ST products used worldwide. They vary greatly in appearance and toxicant emissions and in their composition of tobacco and nontobacco constituents [6]. In Sudan, DT is mixed with an aqueous solution of sodium bicarbonate (locally known as *Atron*) [35]. This constituent may dampen the effect of nicotine on blood pressure. In a previous study conducted about the effects of sodium bicarbonate and sodium chloride on blood pressure and electrolyte homeostasis in normal and hypertensive men, it was found that one month consumption of NaHCO_3_-containing mineral water decreased systolic blood pressure (by 5 mmHg) in the hypertensive subjects [36]. This suggests that the effect of DT is not restricted to tobacco constituents only and nontobacco constituents may exert opposite effects to nicotine. Further studies about the effects of nontobacco constituents of ST on blood pressure are needed.

Increased heart rate after acute exposure to toombak may shift the reflected wave into diastole rather than systole [37], and so a decrease in the augmentation pressure is noted in this study. This finding was confirmed by the linear regression analysis, which revealed an inverse relationship between AP and HR.

Increased myocardial oxygen demand caused by increased heart rate following tobacco dipping should explain the decrease in SEVR. Also, increased heart rate by tobacco dipping should shorten cardiac cycle and explain the decrease noted in ejection duration [38].

## 5. Conclusions

Long-term use of dipping tobacco was not associated with permanent changes in heart rate and blood pressure. Acute exposure to dipping tobacco caused an acute increase in heart rate and oxygen demands of myocardium. Dipping tobacco appears to have a modest effect on cardiovascular risk factors in young healthy men.

Further study with larger sample size and biochemical investigations like epinephrine and norepinephrine levels and using the same dose and type of DT are recommended. We also recommend investigating the effects of first acute exposure to DT in nonusers and various trademarks of *toombak* effects.

## Figures and Tables

**Table 1:** Anthropometric measurements of the study group vs. the control group.

Variable	Study group (*n* = 51)	Control group (*n* = 50)	*P* value
Age (years)	26.9 ± 7.7	27.2 ± 7.1	0.829
Weight (kg)	65.9 ± 13.8	68.9 ± 14.5	0.287
Height (cm)	175.8 ± 6.7	173.6 ± 6.9	0.119
BMI (kg/m^2^)	21.1 ± 4.2	22.8 ± 4.6	0.058

**Table 2:** Central and peripheral hemodynamic parameters for the study group pretobacco dipping vs. the control group.

Variable	Study group (*n* = 51)	Control group (*n* = 50)	*P* value
HR (bpm)	66.15 ± 9.21	72.87 ± 10.13	0.001^∗^
BSP (mmHg)	108.82 ± 9.91	113.16 ± 9.81	0.029
BDP (mmHg)	70.24 ± 8.91	70.80 ± 7.19	0.727
BMP (mmHg)	82.50 ± 6.90	84.25 ± 8.69	0.264
BPP (mmHg)	37.44 ± 8.30	41.57 ± 9.26	0.020
ASP (mmHg)	97.56 ± 9.92	99.31 ± 8.84	0.353
ADP (mmHg)	71.15 ± 8.89	72.19 ± 7.26	0.519
AMP (mmHg)	83.38 ± 8.43	84.86 ± 7.03	0.341
APP (mmHg)	26.58 ± 7.58	27.12 ± 7.36	0.718
AP (mmHg)	4.57 ± 3.65	2.92 ± 2.79	0.012
AIx@75 (%)	14.1 (5.60-21.60)	9.00 (2.87-17.62)	0.080
ED (ms)	279.53 ± 20.47	275.32 ± 20.49	0.304
SEVR%	203.44 ± 30.34	179.11 ± 30.51	0.001^∗^

∗Significant variation. Data were expressed as mean ± SD or median (25^th^-75^th^ interquartile) as applicable.

**Table 3:** Results of linear regression analysis between augmentation pressure and other clinical and PWA parameters.

Variables	Augmentation pressure (AP)		*P* value
	Beta ± SE	95% CI	
Age	0.038 ± 0.021	(-0.021-0.063)	0.316
Weight	−0.040 ± 0.023	(-0.057-0.035)	0.628
BMI	0.030 ± 0.072	(-0.117-0.171)	0.710
HR	−0.242 ± 0.019	(-0.132-0.056)	0.001^∗^
BMP	0.119 ± 0.044	(-0.032-0.143)	0.208
ASP	0.364 ± 0.120	(-0.084-0.394)	0.201
AMP	−0.361 ± 0.133	(-0.450-0.079)	0.166
APP	0.144 ± 0.069	(-0.060-0.214)	0.268
AIx@75	0.685 ± 0.014	(0.221-0.278)	0.001^∗^
ED	0.052 ± 0.009	(-0.008-0.028)	0.275

**Table 4:** Results of PWA in the study group posttobacco dipping vs. the control group.

Variables	Study group Posttobacco dipping (*n* = 51)	Control group (*n* = 50)	*P* value
HR (bpm)	72.50 ± 10.89	72.87 ± 10.13	0.860
BSP (mmHg)	110.16 ± 10.59	113.16 ± 9.81	0.145
BDP (mmHg)	72.06 ± 9.01	70.80 ± 7.19	0.440
BMP (mmHg)	83.98 ± 7.93	84.25 ± 8.69	0.498
BPP (mmHg)	37.01 ± 7.08	41.57 ± 9.26	0.011
ASP (mmHg)	98.20 ± 10.14	99.31 ± 8.84	0.577
ADP (mmHg)	73.13 ± 8.92	72.19 ± 7.26	0.564
AMP (mmHg)	84.89 ± 8.96	84.86 ± 7.03	0.985
APP (mmHg)	24.67 ± 5.83	27.12 ± 7.36	0.182
AP (mmHg)	3.30 (0.60-6.30)	3.00 (1.00-5.50)	0.601
AIx@75%	13.0 (1.60-21.60)	9.00 (2.87-17.6)	0.364
ED (ms)	271.65 ± 19.42	275.32 ± 20.49	0.357
SEVR%	187.11 ± 29.81	179.11 ± 30.51	0.041

Data were expressed as mean ± SD or median (25^th^-75^th^ interquartile) as applicable.

**Table 5:** Results of PWA parameters in the study group pretobacco dipping vs. posttobacco dipping.

Variables	Study group (pretobacco dipping) (*n* = 51)	Study group posttobacco dipping (*n* = 51)	*P* value
HR (bpm)	66.15 ± 9.21	72.50 ± 10.89	0.001^∗^
BSP (mmHg)	108.82 ± 9.91	110.16 ± 10.59	0.181
BDP (mmHg)	70.24 ± 8.91	72.06 ± 9.01	0.053
BMP (mmHg)	82.50 ± 6.90	83.98 ± 7.93	0.079
BPP (mmHg)	37.44 ± 8.30	37.01 ± 7.08	0.658
ASP (mmHg)	97.56 ± 9.92	98.20 ± 10.14	0.508
ADP (mmHg)	71.15 ± 8.89	73.13 ± 8.92	0.033
AMP (mmHg)	83.38 ± 8.43	84.89 ± 8.96	0.071
APP (mmHg)	26.58 ± 7.58	24.67 ± 5.83	0.060
AP (mmHg)	4.30 (2.30-8.00)	3.30 (0.60-6.3)	0.001^∗^
AIx@75%	14.15 (5.60-21.60)	13.0 (1.60-21.60)	0.036
ED (ms)	279.53 ± 20.47	271.65 ± 19.42	0.001^∗^
SEVR%	203.44 ± 30.34	187.11 ± 29.81	0.001^∗^

∗Significant variation. Data were expressed as mean ± SD or median (25^th^-75^th^ interquartile) as applicable.

## Data Availability

The authors confirm that they are ready to provide the study data and materials upon request.
